# Help-Seeking Patterns during Weather Events: 2-1-1 Service Calls among Service-Connected Unhoused Populations in Louisiana from 2014 to 2023

**DOI:** 10.1007/s11524-025-01045-z

**Published:** 2026-02-17

**Authors:** Margaret M. Sugg, Sophia C. Ryan, Michael Erb, Jack Bigelow, Andrew Holbein, Jennifer D. Runkle

**Affiliations:** 1 Department of Geography and Planning, Appalachian State University, P.O. Box 32066, Boone, NC 28608 USA; 2 Research Institute for Environment, Energy and Economics, Appalachian State University, 416 Howard Street, Boone, NC 28608 USA; 3 VIA LINK, 5001 Hwy 190, Suite C-1, Covington, LA 70433 USA; 4 North Carolina Institute for Climate Studies, North Carolina State University, 151 Patton Avenue, Asheville, NC 28801 USA

**Keywords:** Unhoused populations, 2-1-1 service data, Extreme weather, DLNM, Gulf Coast, Extreme temperatures, Cyclones

## Abstract

The 2-1-1 information and referral system connects vulnerable populations to essential services during public health emergencies. However, there is limited evidence examining the specific needs of unhoused populations during these events. This study examines associations between temperature variations, tropical cyclones, and service utilization patterns among unhoused populations using 2-1-1 call data from southern Louisiana (2014–2023). Analyzing over 20,000 calls, predominantly in urban areas (65%), we employed negative binomial regression, distributed lag non-linear modeling (DLNM), and natural language processing to uncover patterns in help-seeking behavior during temperature variations and tropical cyclones. Negative binomial models demonstrate declines in 2-1-1 utilization both during cyclone events (IRR: 0.77, CI: 0.60-0.99) and in the recovery period (IRR: 0.86, CI: 0.76-0.97). Significant associations were observed across the temperature distribution with both the coldest (D1, IRR: = 1.22, 95% CI = 1.08-1.38) and warmest (D10, IRR = 1.21, 95% CI = 1.06-1.38) temperature deciles, indicating increased call volume at the coldest and warmest temperatures compared to moderate temperatures. DLNM results corroborated a U-shaped association at temperature extremes, though effects were predominantly significant only at the coldest temperatures. Computational text analysis of call narratives revealed that mental health mentions increased significantly with temperature (from 19.4% in coldest conditions to 30.5% in warmest conditions), but decreased during cyclone periods (from 29.1% in non-cyclone conditions to 15.5–20.2% during various cyclone phases). While 2-1-1 data captures only those with phone access and service awareness, potentially underrepresenting the most marginalized, these findings provide actionable insights into help-seeking patterns among service-connected (e.g., 2-1-1) unhoused populations during and following extreme weather events.

**Supplementary Information:**

The online version contains supplementary material available at 10.1007/s11524-025-01045-z.

## Introduction

The 2-1-1 information and referral system is a vital lifeline for individuals and families navigating the often complex landscape of health and human services. By simply dialing 2-1-1, callers can quickly connect with community organizations and government agencies that provide essential resources, including food assistance, emergency shelter, mental health support, and disaster relief [1]. Overall, the system serves as “a national safety network for persons to get access readily to assistance” [2], with trained specialists skilled in resource identification and referral. Each year, 2-1-1 call centers handle approximately 20 million calls during public health emergencies, assisting older adults, individuals with disabilities, those experiencing a mental health crisis, and people without stable housing [2, 3].

Despite limitations related to technology access and awareness, 2-1-1 represents one of the most comprehensive and timely datasets on social service needs available nationwide [4, 5]. Previous research has shown that 2-1-1 call data can serve as a valuable early warning system during emerging crises. For example, during Hurricanes Katrina and Rita, 65% of 2-1-1 calls in Texas were related to primary disaster–related unmet needs, including 28% for housing and shelter, 18% for health and safety, and 15% for food and water [6]. Similarly, spikes in calls for housing, food, utilities, and medical assistance occurred during the 2015 Texas floods [7] and Hurricane Irma [4]). In addition to natural disasters, 2-1-1 data has played a crucial role in monitoring public health emergencies, such as the COVID-19 pandemic, with significant increases observed during the pandemic [3].

Despite national recognition and extensive coverage of 2-1-1 services, research on their use during extreme weather events remains limited. As demand for 2-1-1 surges [3], presumably among at-risk populations such as the unhoused, the connection between extreme weather events and the service needs of people experiencing housing instability remains poorly understood. This is a concerning gap in understanding as unhoused individuals face disproportionate exposure to extreme heat, cold, and natural disasters [8]. For instance, analysis of the National Hospital Ambulatory Medical Care Survey has found that unhoused individuals have over four times greater risk of temperature-related illness than the housed [9], and emergency department visits related to homelessness increased after Hurricane Sandy [10]. Yet, preventative measures such as public health messaging often fail to reach these vulnerable sub-populations [11].

Research demonstrates that 2-1-1 effectively reaches socioeconomically disadvantaged populations who are often at lower income and education levels, minority status, and suffer higher rates of unemployment, populations that overlap significantly with those experiencing homelessness [12, 13]. While acknowledging that 2-1-1 data only captures those aware of and able to access the services, potentially underrepresenting individuals without phones or those with severe mental illness, the system’s established infrastructure and trained specialists make it a valuable proxy for understanding service needs among hard-to-reach populations like the unhoused [4, 5, 44]. Additionally, estimates suggest that at least 62% of unhoused youth have cell phones [14], and recent estimates indicate higher rates among youth and adults, ranging from 72 to 94% [15, 45]).

Nonetheless, it is crucial to acknowledge that 2-1-1 data captures a specific subset of the unhoused population, those who are “service-connected,” meaning they have phone access, awareness of 2-1-1 services, and willingness to engage with formal support systems. This population may differ systematically from unhoused individuals who remain disconnected from formal services due to factors including severe, untreated mental illness, lack of consistent phone access, geographic isolation, language barriers, or deliberate avoidance of institutional support based on past negative experiences. While acknowledging these limitations, 2-1-1 represents one of the most comprehensive real-time datasets available for understanding service needs during crises, serving as a critical access point within the broader ecosystem of emergency response that includes emergency departments, shelters, faith-based organizations, and informal community networks [4, 5]. Understanding patterns within this service-connected population is essential for optimizing this specific service pathway while recognizing that it represents one component of a multifaceted support system.

Using 2-1-1 call data as a real-time indicator of service demand provides insight into when, where, and why people seek assistance, particularly during disasters or periods of extreme weather, serving as one of the most comprehensive and timely datasets on social needs [4, 5]. For instance, the potential of 2-1-1 data was most apparent during the COVID-19 pandemic, where real-time data informed understanding of local population needs, including those experiencing economic disruptions or localized COVID-19 outbreaks [4, 5, 16]. By analyzing 2-1-1 call volume, this study examines the relationship between temperatures and tropical cyclones, as well as 2-1-1 service utilization for unmet needs among the unhoused population in southern Louisiana. We employ negative binomial regression to model the association between weather events and service calls, with distributed lag non-linear models (DLNM) as a secondary analysis to capture complex non-linear temperature-response associations. Additionally, we apply computational text mining techniques to analyze narrative content from call records, enabling the systematic identification of service needs and contextual factors that traditional quantitative approaches may overlook during extreme weather events. Identifying patterns in help-seeking behavior can inform targeted interventions, improve resource allocation, and strengthen disaster response strategies to ensure that unhoused individuals receive timely, life-saving support during extreme weather events.

## Methods

### 2-1-1 Data

The United Way 2-1-1 helpline call center is a nationally designated call center that connects clients with needed health and social services in response to expressed needs, including assistance with utilities, rent, mortgage, food, transportation, public services, and medical care assistance [16]. This study obtained call data from 2014 to 2023 from VIA LINK, the 2-1-1 provider for Southern Louisiana, to analyze service requests among the unhoused population. Call records include anonymized timestamps, geographic locations, caller demographics, caller-reported needs (e.g., emergency shelter, cooling centers, utility assistance, medical aid), and narrative statements from the VIA LINK counselor about the call. All 2-1-1 callers provide a zip code location during call intake with the 2-1-1 specialist, with geographic distribution predominantly in southern Louisiana, including the urban areas of New Orleans and Baton Rouge (Supplementary Fig. 1). According to definitions from the Louisiana Department of Health, approximately 65% of calls originated in urban parishes [17]. This population represents a specific subset characterized by phone access, awareness of available services, and engagement with formal support systems.

To contextualize our findings, we compared 2-1-1 caller demographics with HUD’s Homeless Management Information System (HMIS) Stella P reports for the same region (CoC LA-503, 2019). HMIS shelter data documented 3920 individuals in 2019, with higher proportions of Black/African American clients (57.3%, *n* = 2,247 vs. 35.9%, n=7,288) but comparable age distributions for young adults aged 18–24 (11%, *n* = 443 vs. 9.7%, n=1,963) and older adults 65+ (5%, *n* = 206 vs. 6.4%, n=1,297). Unlike HUD’s HMIS system, only 11.4% (*n* = 2,317) of 2-1-1 callers reported staying in emergency shelter the previous night, while 12.3% (*n* = 2,500) were unsheltered in places not meant for habitation. These differences suggest that 2-1-1 reaches individuals across the housing instability spectrum, including those not yet engaged with the shelter system, and therefore is a component of a multifaceted system serving the hard-to-reach population of the unhoused.

### Outcome

The primary outcome of interest was 2-1-1 call volume from service-connected unhoused individuals, identified through demographic information indicating housing status (e.g., “homeless yes-no” designation or “place not meant for habitation” in overnight location fields). The main outcome analyzed was total daily call volume from unhoused individuals across all service types. Secondary outcomes included standardized service need categories recorded by 2-1-1 specialists during intake: housing/shelter, food assistance, and basic needs. Additionally, computational text analysis of 2-1-1 specialist call narratives identified mental health concerns, suicidal ideation, substance use issues, housing/shelter needs, basic needs assistance, and healthcare requirements using predetermined lexicons (Supplemental Table 1).

### Exposure Assessment

Temperature data were obtained from the PRISM Climate Group, which provides daily measurements of mean, minimum, and maximum temperatures, as well as daily precipitation [18]. These data were then averaged to the ZIP Code Tabulation Area (ZCTA) level to correspond with the 2-1-1 data. Tropical cyclone data were compiled from the National Hurricane Center’s Atlantic hurricane database and supplemented with information on landfall locations, timing, and strength classifications (Table 1). We classified days surrounding tropical cyclone events into five periods: pre-cyclone (7 days before landfall), during-cyclone (landfall through 2 days post-landfall), immediate-aftermath (3–14 days post-landfall), acute recovery-period (15–30 days post-landfall), and non-cyclone (all other days). Tropical cyclone periods were applied uniformly across all ZCTAs based on temporal proximity to landfall rather than spatial distance from the storm track.

Temperature associations were examined using two complementary approaches. The primary approach employed decile-based temperature classification to analyze service utilization patterns across the full temperature distribution, dividing temperatures into 10 equal groups (D1–D10), with each decile representing 10% of study days. This approach captures seasonal and temperature-dependent variations in service utilization rather than focusing solely on meteorologically defined temperature extremes. This approach provides readily interpretable incidence rate ratios (IRRs) suitable for informing operational decisions by our community partner, VIA LINK.

The second approach utilized a continuous mean daily temperature within the distributed lag non-linear model (DLNM) framework [46]. This approach better accommodates the small-area case-time series design and captures non-linear temperature-response relationships with distributed lag effects. While not directly comparable to negative binomial results, DLNM provides complementary insights into temperature exposure-response associations at meteorological extremes (5th and 95th percentiles).

### Statistical Analysis

Descriptive statistics were employed to analyze the distribution and frequency of 2-1-1 call data across key variables, including caller demographics, geographic regions, and reported service needs. Statistical significance was assessed at *α* = 0.05.

### Binomial Regression of Cyclones and Temperature

To analyze the overdispersed count data of service calls, we employed negative binomial regression, a well-established method for handling count data where variance exceeds the mean [19]. Negative binomial regression appropriately accounts for the overdispersion commonly found in call volume data, allows the incorporation of multiple predictors, and provides readily interpretable results as incidence rate ratios (IRRs). Our modeling approach analyzed daily aggregated call counts across all ZCTAs without an offset term. This specification focuses on absolute service demand to inform resource allocation during extreme weather events. Temperature associations were modeled using decile-based classification, with the middle two deciles (D5–D6) combined as the reference category representing moderate temperatures (53.2–66.0 °F/11.8–18.9 °C). Cyclone periods were classified into five categories based on temporal proximity to landfall: pre-cyclone (7 days before), during-cyclone (landfall through 2 days post), immediate-aftermath (3–14 days post), recovery-period (15–30 days post), and non-cyclone (reference).

We developed multiple models to examine different aspects of service utilization: (1) temperature deciles and cyclone effects on total 2-1-1 calls, (2) models examining specific service need categories (housing/shelter, food, and basic needs) as coded by 2-1-1 specialists during call intake (i.e., counselor defined needs), and (3) temperature-cyclone interaction models incorporating multiplicative interaction terms, with model comparison via likelihood ratio tests to assess for effect modification.

**Table 1:** Louisiana Hurricanes and Tropical Storms (2014-2023). This table presents the hurricanes and tropical storms that affected Louisiana during the study period, including their dates of landfall or closest approach, strength classifications, and impact descriptions

Name	Date	Strength	Impact Description
Cindy	June 22, 2017	TS	First landfall in LA since 2012
Harvey	August 30, 2017	TS	Made final US landfall, causing flooding
Nate	October 8, 2017	Cat 1	Made landfall in the Mississippi River delta
Barry	July 13, 2019	Cat 1	Made landfall as a minimal Cat 1 with heavy rainfall
Olga	October 26, 2019	TS	Remnants produced 10-15 inches of rain
Cristobal	June 7, 2020	TS	Made landfall east of Grand Isle with a storm surge
Marco	August 24, 2020	TS	Passed south of the Mississippi River delta as a weak TS
Laura	August 27, 2020	Cat 4*	Catastrophic damage in southwest LA with 150mph winds
Delta	October 9, 2020	Cat 2	Followed Laura's path six weeks later
Zeta	October 28, 2020	Cat 3*	Passed directly over New Orleans as a major hurricane
Claudette	June 19, 2021	TS	Formed near Houma with heavy rainfall
Ida	August 29, 2021	Cat 4*	A major hurricane that struck on the anniversary of Hurricane Katrina

*Denotes significant hurricane (Category 3+)

All models included fixed effects for calendar month and year to control for seasonality and long-term trends. Results are presented as IRRs with 95% confidence intervals. All analyses were conducted in R version 4.3.2 using the MASS package for negative binomial regression [47].

### Small-Area Analysis of Extreme Temperatures

As a secondary analysis, we employed the small-area case-time series design using distributed lag non-linear models (DLNM) to investigate the associations between temperature and 2-1-1 calls from the service-connected unhoused [20, 21]. This small-area approach models multiple ZCTA-specific time series without aggregation, accounting for fixed place-based factors. Temperature exposure-response was modeled using natural splines with knots at the 10th, 50th, and 90th percentiles. Seasonal trends were controlled through day-of-year splines (6 degrees of freedom) interacted with year indicators, controlling for long-term (i.e., multi-year) trends. Fixed-effects quasi-Poisson regression with ZCTA-year-month strata accounted for variations in within-area baseline risk. As in previous work, we included day of the week as a categorical variable [22–25]. Model specifications were determined using previous studies as a reference, and model fit was assessed using quasi-AIC [20, 23, 24]. Results were presented at extreme temperature thresholds (i.e., 5th and 95th percentiles) to understand extreme temperatures and 211 utilization. All analyses were conducted using the “dlnm” package [26], with additional support from the “splines” package for natural spline functions, “mgcv” for generalized additive models [27], and “sf” for spatial data handling [28].

### Natural Language Processing Approach

We analyzed call narratives using a lexicon-based approach with six categorical dictionaries comprising: mental health (86 terms), suicidal ideation (22 terms), substance use (58 terms), housing/shelter (45 terms), basic needs (41 terms), and healthcare (76 terms) (Supplemental Table 1). Text preprocessing included case normalization and word boundary detection using regular expressions to ensure whole-word matches [29]. Each narrative was binary coded for the presence/absence of category terms using base R pattern-matching functions. We calculated mention rates by temperature deciles (D1–D10) and cyclone periods. Pearson’s chi-square tests (*α* = 0.05) were used to examine the associations between weather conditions and service category mentions, aiming to determine statistical differences between categories.

## Results

**Fig. 1:**
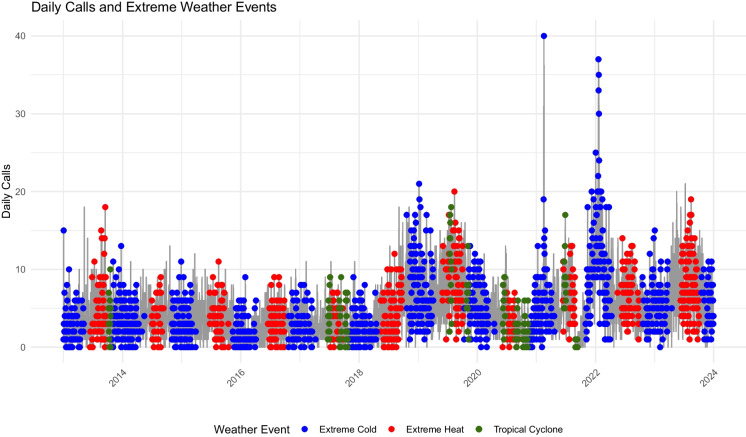
Time series plot showing daily call volumes during extreme cold (blue), extreme heat (red), and tropical cyclone (purple) events. Note the pronounced spike in calls during the 2021 extreme cold event associated with winter storm Uri, with daily call volumes reaching 40 calls.

Table 2 provides demographic characteristics of 2-1-1 service-connected, unhoused callers. A total of 20,303 calls were analyzed across 345 ZIP Code Tabulation Areas (ZCTAs) from January 1, 2014, to December 31, 2023. This region experienced temperatures ranging from −18.0 to 42.7 °C (0 to 108.8 °F). Women constituted the majority (65.3%, *n* = 13,265) of those seeking assistance, while men accounted for 27.9% (*n* = 5671). Most callers were working-age adults (25–64 years), representing 55.0% (*n* = 11,163) of the sample, followed by young adults aged 18–24 (9.7%, *n* = 1963) and older adults aged 65 and older (6.4%, *n* = 1297). Black/African American callers comprised 35.9% (*n* = 7288) of the sample, followed by White/Caucasian callers at 18.6% (*n* = 3768). Figure 1 depicts the total daily call volume during extreme weather events. The most dramatic increase occurred during an extreme cold event in 2021, when calls reached approximately 40 per day related to winter storm Uri.

**Table 2:** Demographics and Service Needs of Unhoused Individuals Using 2-1-1 Services between 2014 and 2023 (N=20,303)

Characteristic	Category	n (%)
Gender	Female/Woman	13,265 (65.3)
	Male/Man	5,671 (27.9)
	Other/Unspecified	1,367 (6.7)
Age	0-12	<10 (0.0)
	13-17	124 (0.6)
	18-24	1,963 (9.7)
	25-64	11,163 (55.0)
	65+	1,297 (6.4)
	Unspecified	5,749 (28.3)
Race	Asian/Middle Eastern	35 (0.2)
	Black/African American	7,288 (35.9)
	Hispanic/Latino	176 (0.9)
	Indigenous/Native	53 (0.3)
	Multiple Races	142 (0.7)
	Unknown/Undisclosed	8,841 (43.5)
	White/Caucasian	3,768 (18.6)
Insurance	Covered	8,526 (42.0)
	Not Covered	3,105 (15.3)
	Unspecified	8,675 (42.7)

### Weather Effects on Service Utilization–Tropical Cyclone Effects

**Fig. 2:**
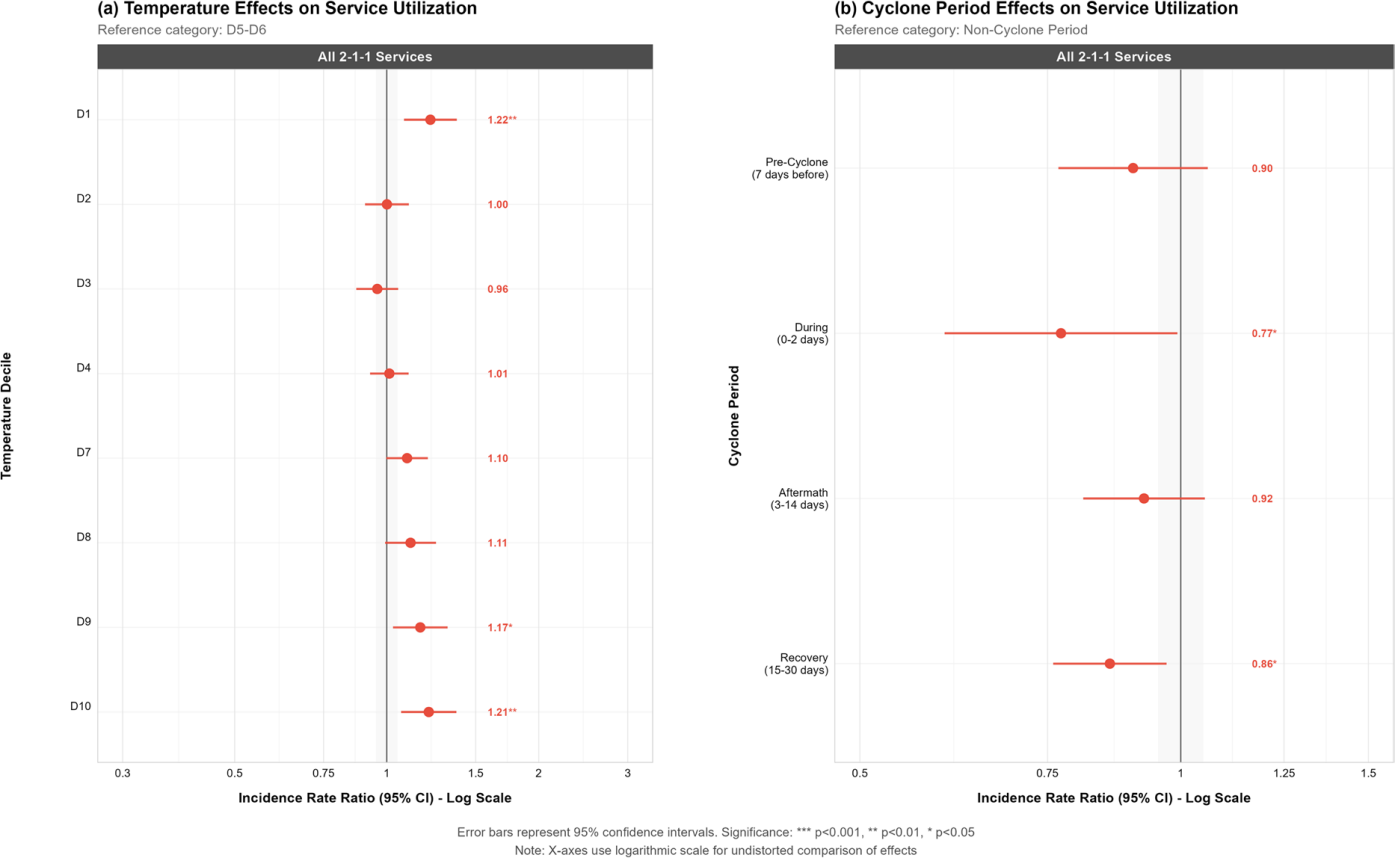
Forest plots displaying incidence rate ratios (IRR) and 95% confidence intervals for weather effects on 2-1-1 service utilization. Panel **a** shows temperature effects across deciles relative to moderate temperatures (D5–D6), with temperature ranges from coldest (D1) to warmest (D10). Panel **b** displays cyclone period effects relative to non-cyclone baseline periods. Error bars represent 95% confidence intervals, with statistical significance indicated by asterisks (***p < 0.001, **p < 0.01, *p < 0.05). X-axes use a logarithmic scale

Cyclone impacts revealed consistent decreases in service utilization across all cyclone periods relative to non-cyclone baseline conditions (Fig. 2b). For total 2-1-1 services, reductions were observed during pre-cyclone periods (IRR = 0.90, 95% CI = 0.77–1.06), during-cyclone periods (IRR = 0.77, 95% CI = 0.60-0.99), immediate-aftermath (IRR = 0.92, 95% CI = 0.81–1.05), and recovery periods (IRR = 0.86, 95% CI = 0.76-–0.97). Significance was observed only for during and recovery periods at p <0.05. Tropical cyclone periods showed no statistically significant effects on any specific service need category (housing, food, basic needs) (Supplemental Fig 2.).

Temperature exposure on 2-1-1 service utilization revealed a U-shaped association, with both cold and hot extremes associated with increased call volumes compared to moderate temperatures (Fig. 2a). The coldest temperature decile (D1) demonstrated the strongest association, with service utilization increasing by 22% (IRR = 1.22, 95% CI = 1.08-1.38 *p *< 0.01) relative to the middle temperature range (D5–D6). The warmest temperatures also showed significant increases, with the hottest decile (D10) associated with a 21% increase in calls (IRR = 1.21, 95% CI = 1.06-1.38, *p *< 0.01) and the second-warmest decile (D9) showing an 17% increase (IRR = 1.17, 95% CI = 1.02-1.36).

Analysis revealed significant associations between cold temperatures and increased service need mentions, with housing/shelter requests rising 71% (IRR = 1.71, 95% CI = 1.42–2.05), food needs increasing 50% (IRR = 1.50, 95% CI = 1.01–2.23), and basic needs mentions rising 64% (IRR = 1.64, 95% CI = 1.13–2.37) during the coldest temperature decile compared to moderate conditions (Supplemental Fig 3.). Higher temperatures showed more limited associations, with housing/shelter mentions increasing significantly by 37% (IRR = 1.37, 95% CI = 1.13–1.67) in the hottest decile, while food and basic needs showed non-significant increases (IRR = 1.34, 95% CI = 0.85–2.10 and IRR = 1.38, 95% CI = 0.90–2.12, respectively) in warmer weather (Supplemental Fig 3.). A temperature-cyclone interaction analysis revealed significant effect modification (χ² = 56.91, p = 0.0015), with interaction ratios ranging from 0.24 to 1.54 (Supplemental Figure 4). Four statistically significant interactions (p < 0.1) showed negative departures from multiplicative expectations: D3 during immediate-aftermath periods (ratio = 0.56, p < 0.05), D8 during immediate-aftermath periods (ratio = 0.35, p < 0.05), D9 during immediate-aftermath periods (ratio = 0.29, p < 0.001) and D3 during recovery periods (ratio = 0.40, p < 0.1). One notable positive interaction occurred at D2 temperatures during active cyclone periods (ratio = 1.54), though this was not statistically significant.

**Fig. 3:**
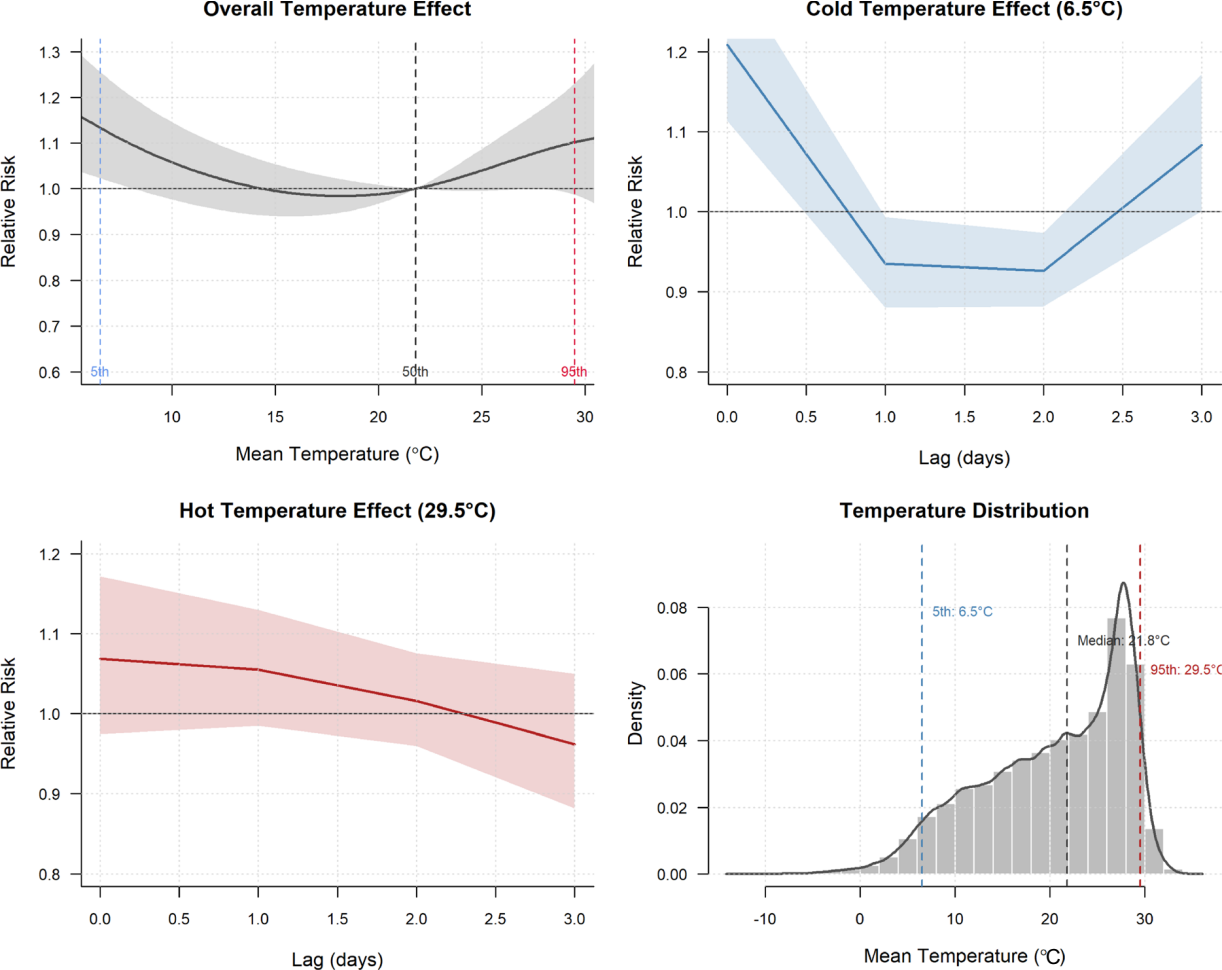
Distributed lag non-linear model (DLNM) results showing **A** overall cumulative relationship between mean temperature (°C) and relative risk (RR) of homelessness service calls with 95% confidence intervals, reference at median temperature, and vertical lines indicating 5th, 50th, and 95th percentile temperatures; **B** lag-specific effects at the 5th percentile (6.5 °C) of temperature over 3 days, demonstrating immediate high risk followed by rapid decline; **C** lag-specific effects at the 95th percentile showing moderately elevated risk that attenuates gradually; and **D** temperature distribution with density curve and markers for key percentiles

### DLNM

The overall temperature-response curve demonstrated that temperatures at both extremes were associated with elevated risks of service calls compared to the median temperature. Results indicate a non-linear association with elevated risk at temperature extremes (U-shaped curve), and differential temporal patterns between cold and hot exposure effects (Fig. 3). Both the 5th percentile temperature and the 95th percentile temperature  exhibit elevated incidence risk ratios, although the effect appears more significant at colder temperatures.

**Fig. 4:**
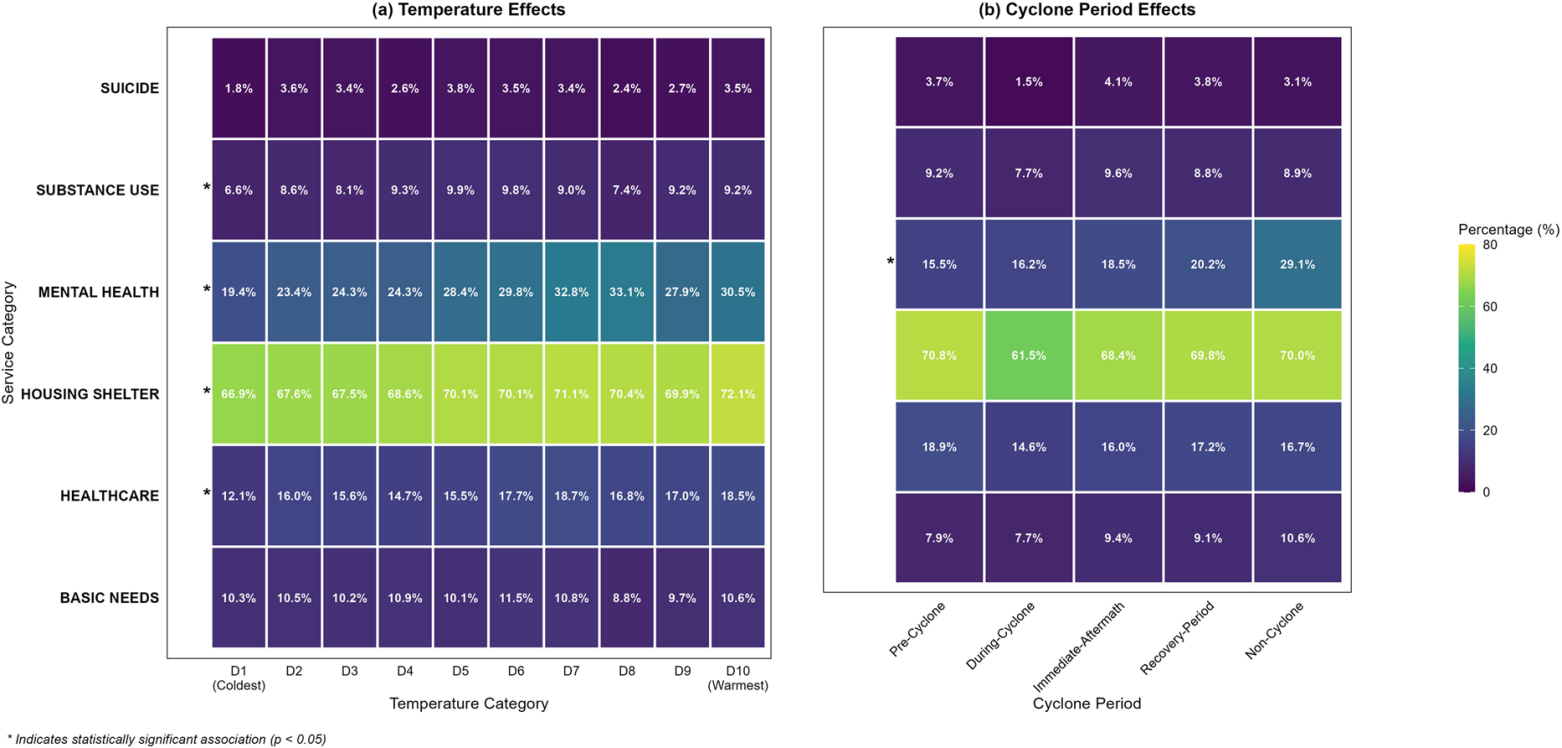
Computational text analysis of 2-1-1 call note narratives using predefined service category lexicons (Supplemental Table 1). Panel **a** shows service mention frequencies across temperature deciles from coldest (D1) to warmest (D10) conditions. Panel **b** displays service mention patterns across five cyclone periods: pre-cyclone (7 days before landfall), during-cyclone (landfall through 2 days after), immediate-aftermath (3–14 days after), recovery-period (15–30 days after), and non-cyclone periods. Percentages indicate the proportion of calls mentioning each service category. Asterisks (*) denote statistically significant associations (*p* < 0.05) based on chi-square independence tests. Mental health, substance use, housing/shelter, and healthcare mentions showed significant temperature effects, while only mental health mentions varied significantly across cyclone periods

### Natural Language Processing

Figure 4 illustrates the comparative patterns of service category mentions from computational text analysis with predetermined lexicons (Supplemental Table 1) across weather conditions. Temperature showed significant associations for multiple service categories (Table 3). Mental health mentions generally increased from 19.4% in the coldest decile (D1) to 30.5% in the warmest decile (D10, *p* < 0.001), with healthcare mentions following a similar pattern from 12.1 to 18.5% (*p* < 0.001). Housing/shelter needs also increased significantly with temperature from 66.9 to 72.1% (*p* = 0.003), while substance use mentions showed significant but more variable patterns across temperature deciles (*p* = 0.041). Basic needs mentions were more stable; approximately 10% across all temperature conditions (*p* = 0.453).

**Table 3:** Percentage of Calls Mentioning Service Categories by Temperature Quantiles in Louisiana, 2014-2023

	Temperature Deciles (Q1 = Coldest 10% → Q10 = Warmest 10%)										
NLP Category	D1	D2	D3	D4	D5	D6	D7	D8	D9	D10	*P*-value
Mental Health	19.4%	23.4%	24.3%	24.3%	28.4%	29.8%	32.8%	33.1%	27.9%	30.5%	*p* < 0.001***
Suicide	1.8%	3.6%	3.4%	2.6%	3.8%	3.5%	3.4%	2.4%	2.7%	3.5%	*p* = 0.028*
Substance Use	6.6%	8.6%	8.1%	9.3%	9.9%	9.8%	9.0%	7.4%	9.2%	9.2%	*p* = 0.041*
Housing Shelter	66.9%	67.6%	67.5%	68.6%	70.1%	70.1%	71.1%	70.4%	69.9%	72.1%	*p* = 0.003**
Basic Needs	10.3%	10.5%	10.2%	10.9%	10.1%	11.5%	10.8%	8.8%	9.7%	10.6%	*p* = 0.453
Healthcare	12.1%	16.0%	15.6%	14.7%	15.5%	17.7%	18.7%	16.8%	17.0%	18.5%	*p* < 0.001***

Q1 represents the coldest 10% of days, Q10 represents the warmest 10% of days. Percentages indicate the proportion of calls mentioning each service category. Note: *p < 0.05, **p < 0.01, ***p < 0.001. Statistical significance was assessed using chi-square independence tests to determine whether service category mentions varied across temperature conditions. Overall *p*-values are reported for each service category

Cyclone period analysis revealed that only mentions of mental health varied significantly across storm phases (Table 4). Mental health mentions were substantially lower during all cyclone-related periods compared to non-cyclone conditions: pre-cyclone (15.5%), during-cyclone (16.2%), immediate-aftermath (18.5%), and recovery periods (20.2%) versus non-cyclone periods (29.1%, *p* < 0.001). All other service categories showed non-significant variations.

**Table 4:** Percentage of Calls Mentioning Service Categories by Cyclone Period

	Cyclone Period					
Category	Pre-Cyclone	During-Cyclone	Immediate-Aftermath	Recovery-Period	Non-Cyclone	*P*-value
Mental Health	15.5%	16.2%	18.5%	20.2%	29.1%	*p* < 0.001***
Suicide	3.7%	1.5%	4.1%	3.8%	3.1%	*p* = 0.349
Substance Use	9.2%	7.7%	9.6%	8.8%	8.9%	*p* = 0.957
Housing Shelter	70.8%	61.5%	68.4%	69.8%	70.0%	*p* = 0.273
Basic Needs	7.9%	7.7%	9.4%	9.1%	10.6%	*p* = 0.208
Healthcare	18.9%	14.6%	16.0%	17.2%	16.7%	*p* = 0.711

Percentages indicate the proportion of calls mentioning each service category. Note: *p < 0.05, **p < 0.01, ***p < 0.001. Statistical significance was assessed using chi-square independence tests to determine whether service category mentions varied across cyclone conditions. Overall *p*-values are reported for each service category

## Discussion

This study examined the association between weather events and service utilization among unhoused populations using 2-1-1 call data in southern Louisiana from 2014 to 2023. By analyzing over 20,000 calls, we identified notable patterns in help-seeking behavior among 2-1-1 users and different weather events. While temperatures were significantly associated with changes in service utilization, consistent declines, with reductions during active cyclone events and recovery periods during tropical cyclones, possibly due to power outages, disrupted networks, or increased utilization of other resources (e.g., shelters, emergency services), highlighting a complex response pattern. This finding challenges conventional assumptions about disaster response. It suggests the presence of multiple barriers, including disrupted communication infrastructure, evacuation dynamics, or strategic reliance on alternative support networks, which may influence the utilization of 2-1-1 among the unhoused. In contrast, temperatures exhibited consistent associations, particularly at the highest and lowest temperatures, with more 2-1-1 calls related to housing and shelter concerns. These results have implications for disaster preparedness and response strategies targeting service-utilizing unhoused populations, who face disproportionate exposure to climatic events [8, 9] and simultaneously encounter structural barriers to accessing traditional support systems [10, 11].

We observed reductions in 2-1-1 call volume during active cyclone events and recovery periods, with non-significant trends during pre-cyclone and immediate-aftermath phases. Beyond individual decision-making, systemic factors may create additional barriers to 2-1-1 access during cyclones, including power outages, telecommunications disruptions, and cell tower damage that physically prevent calling. During declared emergencies, alternative service pathways typically become available that are not accessible during non-disaster periods. Emergency shelters open with fewer barriers to entry, and emergency services are more actively deployed in affected areas (HUD [30]). Additionally, disasters often activate informal community support networks, including family, friends, faith communities, and neighborhood connections, which provide temporary assistance without requiring formal service systems [31]. Temporary expansion of the safety net may also reduce reliance on 2-1-1 as the primary point of access to services.

Additional explanations may account for this phenomenon. Doran et al. [10] found that emergency department visits for newly unhoused individuals increased following Hurricane Sandy. This suggests that individuals with recently disrupted housing may turn to medical facilities rather than social services during disasters, possibly due to limited awareness of alternative services. Complementing this perspective, Settembrino and Allen [32] documented how chronically homeless individuals often develop sophisticated self-reliance strategies through years of negotiating life on the economic margins. Their qualitative research revealed that many unhoused individuals deliberately choose to “ride out” hurricanes in improvised shelters (i.e., tents) rather than engage with formal emergency services, reflecting lived experience and a preference for autonomy over perceived institutional restrictions. This finding suggests that unhoused populations often assess and respond to risk in dynamic ways [33, 51], which may help explain reduced 2-1-1 call volume during cyclone periods in this marginalized population.

Our temperature analysis revealed a pronounced U-shaped association between weather extremes and service utilization, with both the coldest and warmest temperature deciles showing significantly increased 2-1-1 calls compared to moderate conditions. The distributed lag non-linear modeling corroborated this U-shaped temperature-response pattern. The association was more pronounced during extreme cold, with significantly elevated housing and shelter requests. This pattern is consistent with documented temperature-related health vulnerabilities among unhoused populations [9, 34] and suggests that temperature variations may influence service-seeking behavior among those connected to formal support systems and the need for shelter during these temperatures.

Our computational text analysis of call narratives revealed associations between weather conditions and specific service needs among unhoused populations. Mental health mentions increased as temperatures rose from the coldest to the warmest deciles, while showing significant decreases during cyclone periods compared to non-cyclone conditions. Similarly, rising temperatures were generally associated with increased mentions of housing/shelter, as well as healthcare concerns. These temperature-dependent patterns suggest shifts in seasonal vulnerability [34], while the cyclone-related decrease in mental health mentions, despite consistent proportions of other service needs, indicates a potential reprioritization of immediate survival needs during disaster phases. Our findings highlight how climate conditions shape the volume and content of help-seeking behavior, with implications for seasonally tailored intervention strategies that address these shifting priority landscapes.

The demographic profile of unhoused 2-1-1 callers, predominantly women (65.3%) and working-age adults (55.0%), reveals complex social dynamics underlying service utilization patterns beyond simple exposure risks, and aligns with general 2-1-1 utilization patterns, whereby adult women account for the majority of 2-1-1 clients [4, 5, 35–37]. These patterns also align with [48] observation that homelessness represents a social position where vulnerability factors intersect, with ethnicity, gender, health, and sexuality creating nuanced risk profiles. The higher proportion of female callers reflects documented gendered differences in help-seeking behavior, potentially driven by caregiving responsibilities, heightened exposure to gender-based violence, or more extensive social networks [38]. Future work incorporating intersectional analysis may demonstrate how help-seeking patterns are shaped by complex survival strategies that challenge simplistic narratives of vulnerability during disaster contexts [39].

### Theoretical and Policy Implications

Traditional disaster response frameworks often overlook the complex realities faced by marginalized populations, particularly those experiencing homelessness, whose risk is shaped more by social, economic, and political conditions than by natural hazards alone [39]. While models such as the Protective Action Decision-Making framework [32, 49, 50] provide valuable insights into threat response, they may not fully capture the decision-making processes of individuals living in chronic precarity [50]. Rather than reflecting passive vulnerability, reduced service utilization during extreme weather events illustrates the agency of unhoused individuals navigating structural barriers to safety [40, 41]. Our findings reinforce Settembrino and Allen’s [32] call to move beyond top-down notions of vulnerability by recognizing localized, experience-driven interpretations of risk [51]. For individuals experiencing homelessness, immediate survival needs often outweigh concerns about weather events, highlighting the need for risk frameworks that center on structural and situational constraints. Policy implications include developing seasonally tailored interventions for temperature-dependent mental health and housing needs, reducing access barriers through non-traditional outreach, investing in year-round resilience efforts, and coordinating community-informed services [32, 51].

### Optimizing 2-1-1 as a Critical Service Pathway

Our findings have direct implications for enhancing 2-1-1 services as one critical access point within the broader emergency response infrastructure. The temperature-dependent patterns we observed, particularly the U-shaped association with increased calls during both extreme cold and heat, suggest opportunities for proactive resource allocation and targeted outreach by 2-1-1 providers during forecasted temperature extremes. The distinct patterns observed during tropical cyclones, showing decreased call volume despite presumably elevated need, highlight the importance of multimodal service delivery that does not rely solely on phone-based systems during disasters, when infrastructure disruptions may limit 2-1-1 accessibility. For 2-1-1 programs specifically, these findings suggest several operational improvements: (1) implementing weather-triggered surge staffing 2-1-1 protocols during temperature extremes; (2) developing partnerships with emergency shelters and emergency services to create seamless referral pathways during cyclone events when 2-1-1 access may be limited; (3) tailoring resource databases to reflect seasonal shifts in service needs, particularly the increased mental health mentions during warmer temperatures; and (4) recognizing that 2-1-1 serves as one node in a larger network that includes emergency departments, homeless shelters, faith-based organizations, and informal community support systems. Understanding utilization patterns among service-connected individuals enables 2-1-1 programs to better serve this population while acknowledging that parallel efforts are needed to reach those not currently connected to formal services.

### Strengths and Limitations

Our study offers several methodological contributions to understanding the relationship between extreme weather events and service utilization among unhoused populations. First, utilizing 2-1-1 call data from a decade (2014–2023) provides a longitudinal perspective on help-seeking behavior during weather events. This approach addresses traditional limitations in homelessness research, which often rely on cross-sectional data collection that fails to capture temporal dynamics [8]. Additionally, real-time 2-1-1 call data to reflect service needs represents an innovative approach to monitoring vulnerable populations during environmental conditions. Unlike traditional surveillance systems, which often experience significant reporting delays, 2-1-1 data captures immediate help-seeking behavior, enabling more responsive intervention strategies during emerging crises [3]. Second, our mixed-methods approach, combining negative binomial regression, distributed lag non-linear modeling, and natural language processing, provides a more comprehensive understanding of service utilization than previous studies. The integration of statistical modeling with computational text analysis of narrative call records offers both quantitative trends and qualitative insights into the lived experiences of unhoused individuals during extreme weather events, addressing the shortcomings of single-method approaches that may overlook critical contextual factors [42].

Despite these strengths, some limitations should be considered. First, 2-1-1 data only captures those aware of and able to access telephone services, potentially underrepresenting the most vulnerable populations, such as individuals without phones, those unaware of 2-1-1 services, or those with severe mental illnesses [3, 43]. Our administrative data also limits differentiation between sub-populations of homelessness, which may experience weather differently. Secondly, temperature exposure assignment may introduce misclassification, as unhoused individuals may be more mobile than housed populations, and their reported ZIP code location may not reflect where they experienced the day’s weather conditions. While this method provides the best available approximation given the constraints of 2-1-1 specialist-collected data, the potential for exposure misclassification should be considered when interpreting the results. Exposure misclassification may also occur from cyclone exposure which was assigned uniformly across the entire study area rather than ZCTA-specifically, which may not capture spatial variations in storm impacts across our study region Thirdly, the geographic scope of our study was limited to southern Louisiana and a limited number of tropical cyclone events (Table 1), which may limit the generalizability of our findings to other regions. Fourth, societal changes over the study period, such as policy shifts and evolving awareness of 2-1-1 services, may confound observed trends. We also cannot determine what proportion of the unhoused population is familiar with 2-1-1 services or likely to use them, which affects the interpretation and generalizability of our findings. Fifth, NLP narrative analysis and counselor-coded categories capture distinct dimensions of service needs, and unadjusted versus adjusted estimates may differ due to confounding potential explaining differences in our results. Finally, while we identify associations between weather and service utilization, causal inferences remain limited due to the potential presence of unobserved variables that influence help-seeking behavior. Future research should integrate primary data collection and comparative geographic analyses to enhance generalizability and address these limitations, and should include other data sources, such as the Department of Housing and Urban Development’s Continuum of Care Program shelter reports, to validate 2-1-1 as a proxy for vulnerable populations, such as the unhoused.

## Conclusions

This study examined help-seeking patterns among service-connected unhoused populations during weather events using nearly a decade of 2-1-1 call data from southern Louisiana. While high and low temperatures significantly increased service utilization, tropical cyclones were associated with reductions in calls during active events and recovery periods, suggesting potential barriers to access or alternative coping strategies during acute disasters. Natural language processing revealed that mentions of mental health increased with temperature but decreased during cyclones, indicating a shifting priority hierarchy. However, findings are limited to service-connected individuals with phone access and system awareness, potentially representing only a subset of the broader unhoused population. Findings suggest that disaster response frameworks should move beyond traditional vulnerability models to recognize the complex decision-making processes of unhoused individuals, incorporating both structural constraints and community-informed approaches to develop seasonally responsive intervention strategies.

## Supplementary Information

Below is the link to the electronic supplementary material.

ESM 1 (DOCX 677 KB)

## Data Availability

The 2-1-1 call data analyzed in this study are not publicly available due to privacy protections for vulnerable populations. Aggregated data supporting the findings may be available from VIA LINK upon reasonable request and with appropriate data use agreements.
