# Supplementary material for: Help-Seeking Patterns during Weather Events: 2-1-1 Service Calls among Service-Connected Unhoused Populations in Louisiana from 2014 to 2023
Source: J Urban Health. 2026 Feb 17;103(1):157–71. doi: 10.1007/s11524-025-01045-z (PMC13136432; doi:10.1007/s11524-025-01045-z)
Supplement: Supplementary file 1 — (DOCX 677 KB) [file 11524_2025_1045_MOESM1_ESM.docx]

**
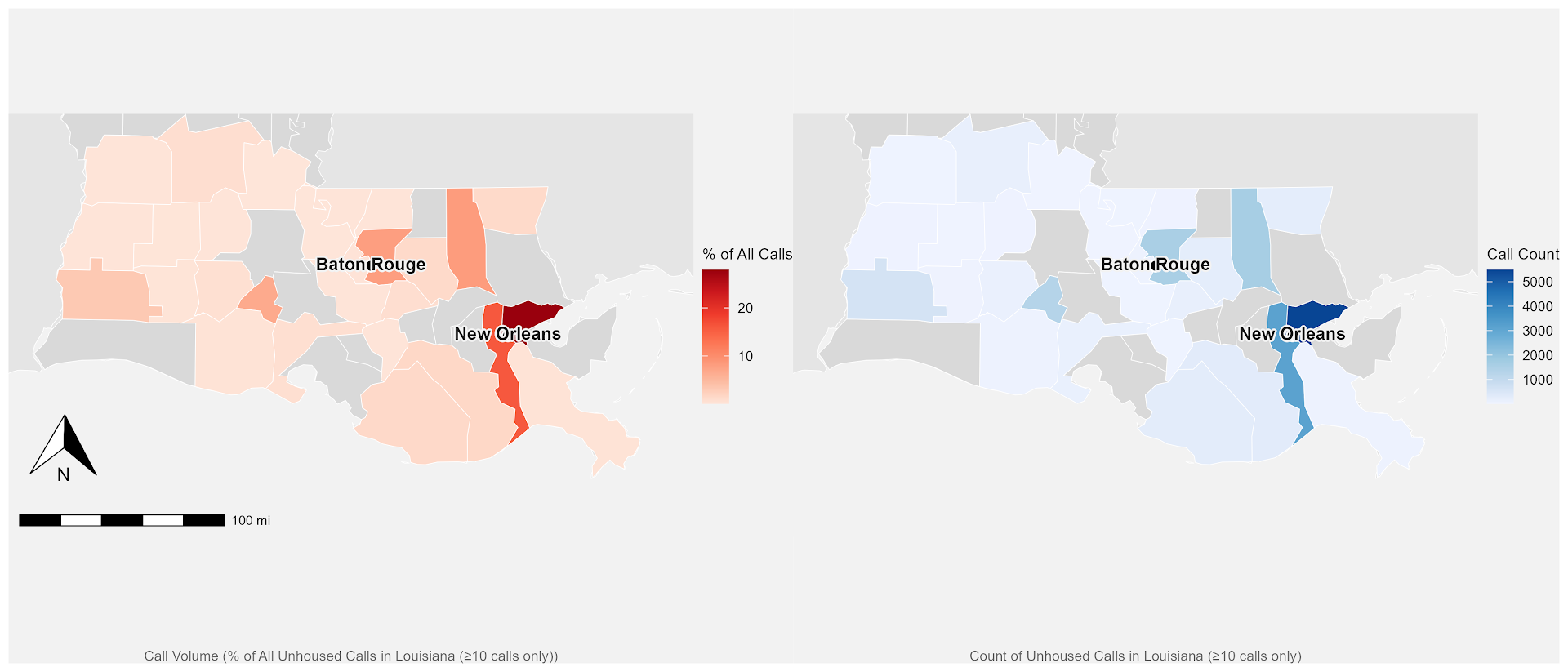
**

**Supplemental Figure 1:** Geographic distribution of 2-1-1 call density across southern Louisiana from 2014 to 2023. Areas with the highest service utilization are concentrated in urban parishes, particularly around New Orleans and Baton Rouge metropolitan areas. The majority of calls (64.5%) originated from parishes classified as urban by the Louisiana Department of Health

**
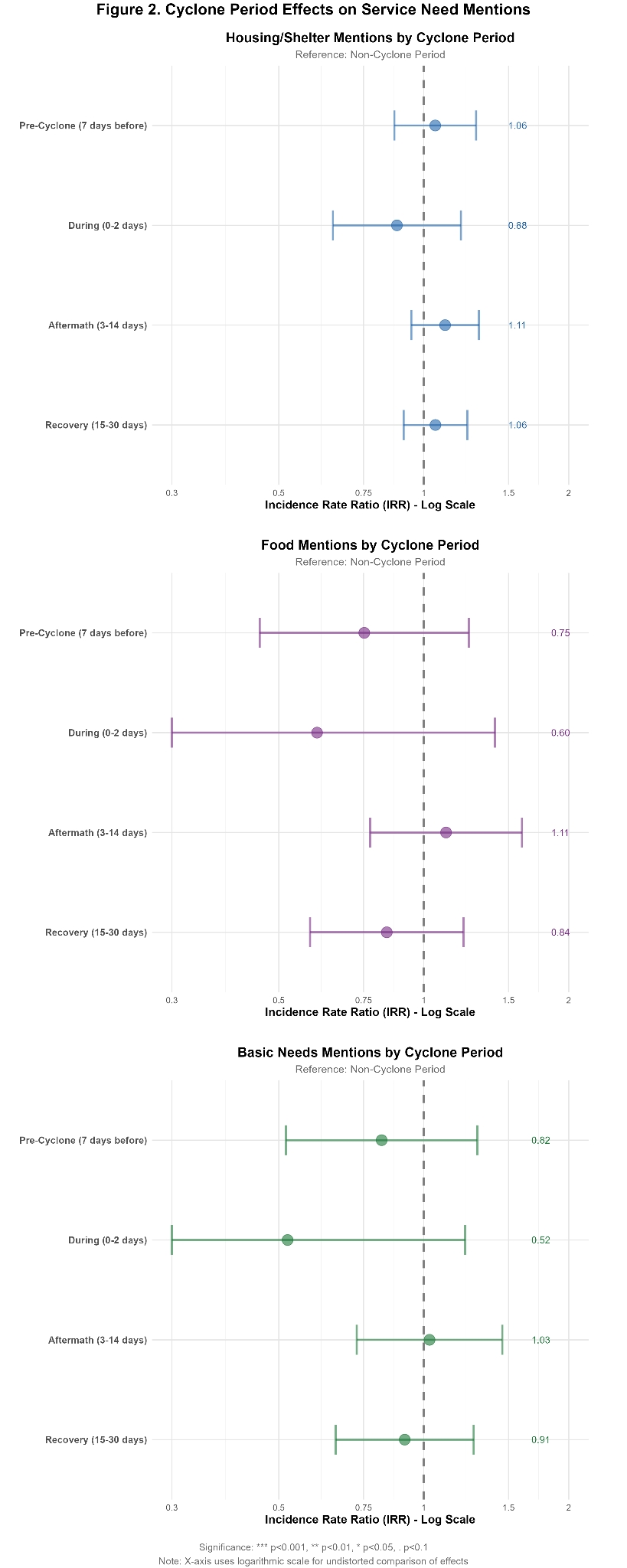
**

**Supplemental Figure 2:** Cyclone Period Effects on Service Need Mentions in 2-1-1 Calls. Counselors identified service needs for this analysis. Forest plots showing incidence rate ratios (IRR) for mentions of different service categories across cyclone periods compared to non-cyclone periods as reference. Three panels display: (A) Housing/Shelter mentions (blue), (B) Food mentions (purple), and (C) Basic Needs mentions (green). Cyclone periods include Pre-Cyclone (7 days before), During (0-2 days), Aftermath (3-14 days), and Recovery (15-30 days). The X-axis uses a log scale for IRR values. Error bars represent 95% confidence intervals.

**
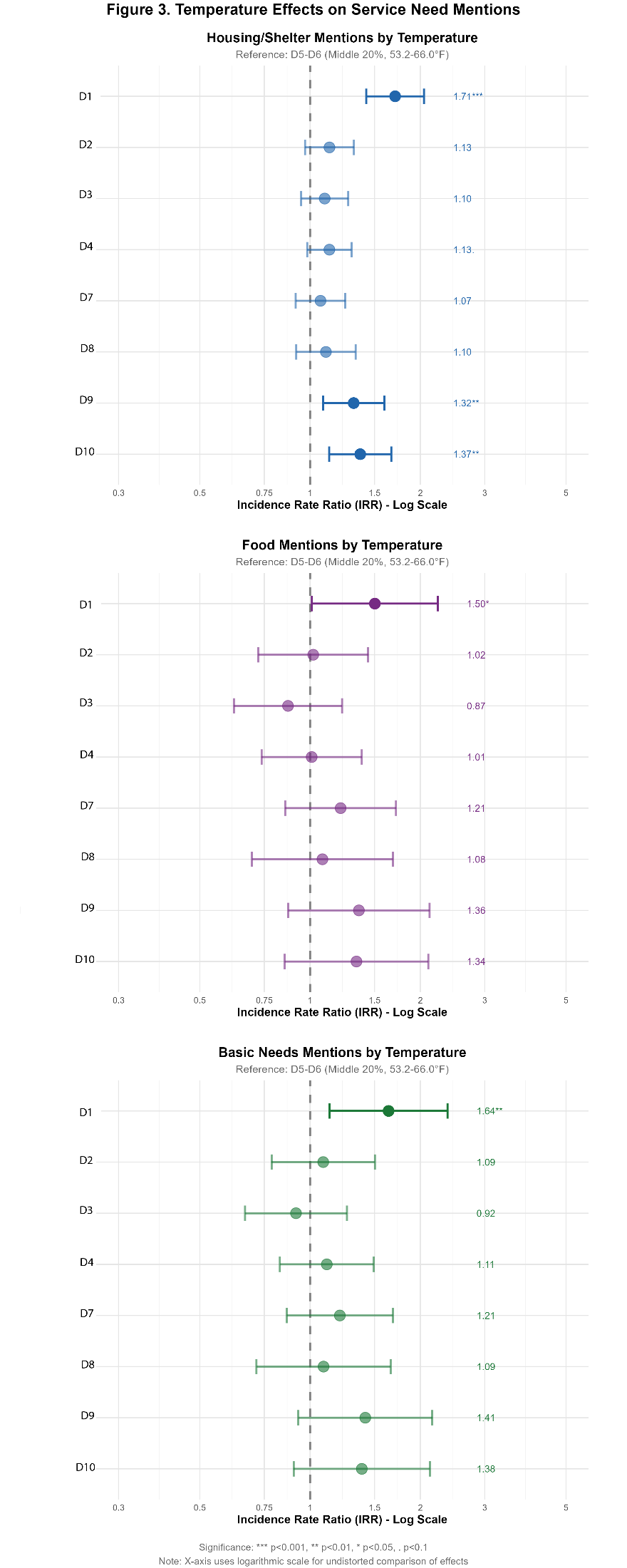
**

**Supplemental Figure 3:** Temperature Effects on Service Need Mentions in 2-1-1 Call. Counselors identified service needs for this analysis. Forest plots showing incidence rate ratios (IRR) for mentions of different service categories across temperature deciles (D1-D10) compared to the reference category (D5-D6). Three panels display: (A) Housing/Shelter mentions (blue), (B) Food mentions (purple), and (C) Basic Needs mentions (green). The X-axis uses a log scale for IRR values. Error bars represent 95% confidence intervals, with asterisks indicating statistical significance (*** p<0.001, ** p<0.01, * p<0.05).

**
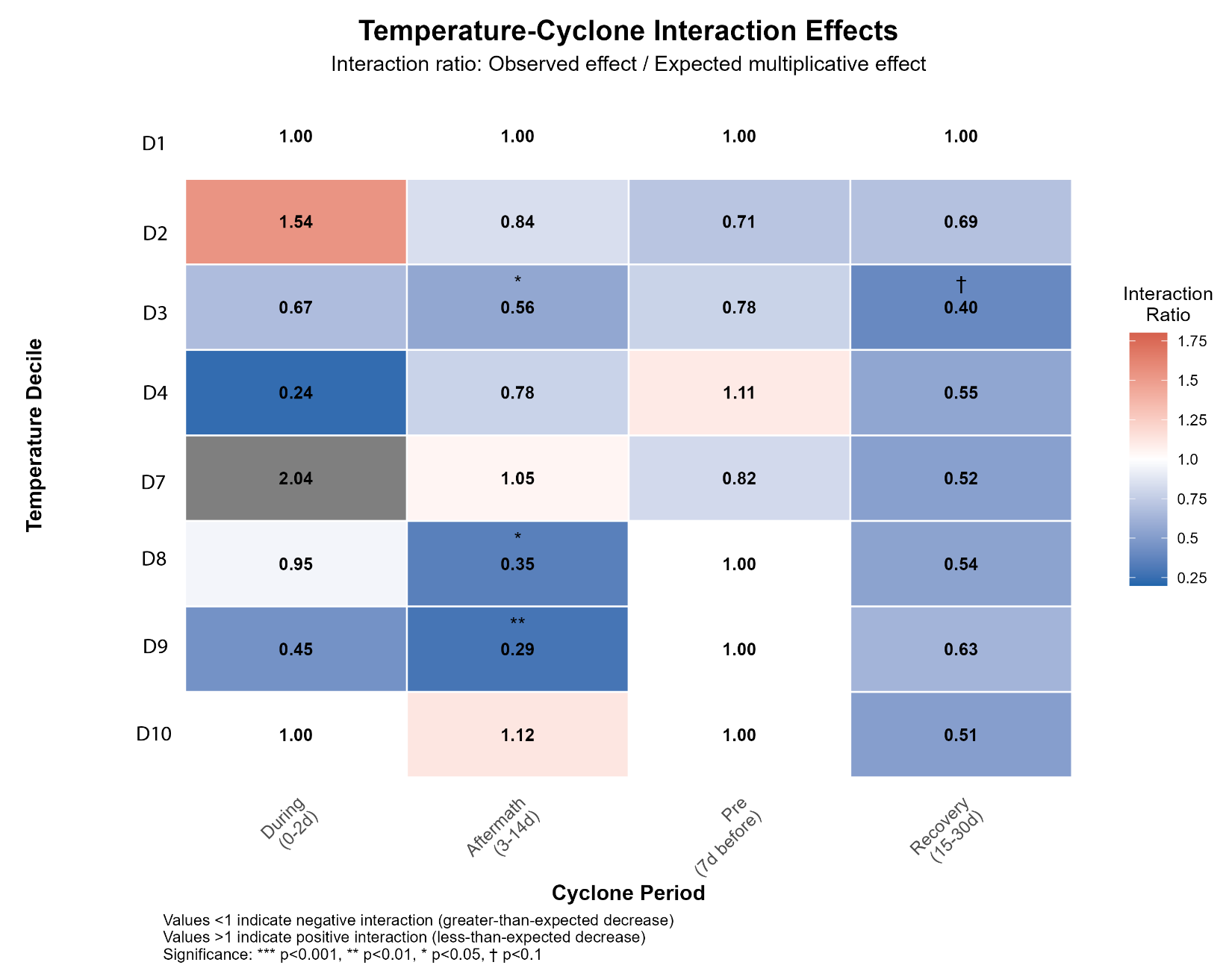
**

**Supplemental Figure 4** Temperature-Cyclone Interaction Effects on 2-1-1 Call Volume. Heatmap showing interaction ratios (observed effect / expected multiplicative effect) across temperature deciles and cyclone periods. Values >1 indicate positive interaction (less suppression than expected), values <1 indicate negative interaction (greater suppression than expected). Temperature deciles D2-D10 are shown relative to reference D5, across cyclone periods: During (0-2d), Aftermath (3- 14d), Pre (7d before), and Recovery (15- 30d). Statistical significance: *** p<0.001, ** p<0.01, * p<0.05, † p<0.1. Notably significant negative interactions occur at cooler temperatures (D3-D4) during aftermath periods and warmer temperatures (D8) during aftermath and recovery periods. Temperature deciles were calculated for each analysis separately.

**Supplemental Table 1:** Comprehensive Natural Language Processing (NLP) Lexicon for Analyzing 2-1-1 Homeless Service Call Narratives from the counselor call notes. Detailed breakdown of terminology used in computational text analysis of homeless service call narratives, categorized by service domain and semantic content.

| Category | Subcategory | All Terms |
| --- | --- | --- |
| Mental Health | Core Terms | anxiety, depression, stress, trauma, crisis, mental, psychiatric, emotional, distress, therapy, counseling, medication, disorder, schizophrenia, bipolar, psychosis, insomnia, panic, afraid, fear, nervous, worry, paranoid, hallucinating, voices, delusion, ptsd, ocd, adhd, dementia, alzheimer, anxious |
|  | Conditions | mental illness, mental health, mental breakdown, psychological, ptsd, ocd, adhd, dementia, alzheimer, anxious, depressed, manic, mania, mood disorder, behavioral health, cognitive, mood swings, irritable, agitated, restless |
|  | Symptoms | nervous, panic, afraid, fear, worry, paranoid, hallucinating, voices, delusion, insomnia, nightmares, flashback, disoriented, confused, irritable, agitated, restless, overwhelmed, can't sleep, no sleep, not sleeping |
|  | Treatment | therapist, counselor, psychiatrist, psychologist, prescription, treatment, support group, therapy session, counseling session, group therapy, inpatient, outpatient, psychiatric hospital, crisis center |
|  | Substance-Related | substance, substance abuse, substance use, addiction, addicted, alcohol, alcoholic, alcoholism, drug, drugs, overdose, withdrawal, detox, rehab, sober, using, heroin, cocaine |
| Suicide | All Terms | suicide, suicidal, kill myself, end my life, take my life, self harm, self-harm, cutting, hurt myself, harming myself, die, death, no reason to live, better off dead, can't go on, cant go on, overdose, give up, hopeless, helpless, wish I was dead, wish I were dead |
| Substance Use | General Terms | substance, substance abuse, substance use, addiction, addicted, withdrawal, detox, detoxification, rehab, rehabilitation, recovery, relapse, sobriety, sober, using, user |
|  | Alcohol-Related | alcohol, alcoholic, alcoholism, drinking, drunk, intoxicated, beer, wine, liquor, binge, hangover |
|  | Drug-Related | drug, drugs, overdose, high, withdrawal, clean, dealer, narcotic, controlled substance, heroin, meth, methamphetamine, cocaine, crack, opioid, opioids, fentanyl, pills, prescription drugs, marijuana, weed, pot, cannabis |
|  | Treatment | syringe, needle exchange, narcan, naloxone, medication assisted, counseling, 12 step, aa, na |
| Housing/Shelter | Emergency Shelter | shelter, emergency shelter, warming center, cooling center, overnight, bed, cot, place to sleep, place to stay |
|  | Housing Terms | housing, apartment, rent, eviction, evicted, landlord, lease, deposit, section 8, public housing, affordable housing, transitional housing, supportive housing, permanent housing, rapid rehousing, home, mortgage, foreclosure |
|  | Homelessness Terms | homeless, homelessness, unhoused, unsheltered, street, sleeping outside, sleeping in car, living in car, tent, encampment, camp, camping |
|  | Housing Barriers | background check, criminal record, felony, credit check, credit score, income verification |
| Basic Needs | Food | food, hungry, hunger, meal, meals, eat, food pantry, food bank, soup kitchen, groceries, snap, ebt, food stamps |
|  | Water | water, thirsty, dehydrated, drinking water, hydration |
|  | Clothing | clothes, clothing, coat, jacket, shoes, boots, socks, blanket, sleeping bag, winter clothes, dress, dressed, outfit |
|  | Hygiene | shower, bath, clean, hygiene, soap, shampoo, toothbrush, toothpaste, deodorant, sanitation |
| Healthcare | General Terms | health, healthcare, medical, doctor, physician, hospital, emergency room, ER, clinic, appointment, exam, screening, diagnosis |
|  | Treatment | treatment, prescription, medicine, medication, pharmacy, pharmacist, refill, injection, shot, procedure, operation |
|  | Insurance | insurance, Medicaid, Medicare, covered, coverage, copay, deductible, uninsured, self-pay, sliding scale, free clinic |
|  | Physical Conditions | pain, chronic, disease, condition, sick, ill, injured, injury, diabetes, hypertension, heart, cardiac, asthma, breathing |
|  | Specific Conditions | tumor, infection, virus, bacteria, covid, coronavirus, flu, pneumonia, std, sti, hiv, aids |
|  | Disability | disability, disabled, handicap, handicapped, mobility, wheelchair, walker, cane, hearing aid, glasses |
|  | Sensory Health | blind, deaf, hard of hearing, sight, vision |

Footnotes:

1. Terms were extracted from 2-1-1 call narratives using computational natural language processing techniques.
2. Lexicon development involves the systematic categorization of service-related terminology with linguistic detection.
3. Case-insensitive matching was employed to ensure comprehensive term detection.
4. Terms represent semantic domains relevant to homeless service needs.
